# Survey of radiological optimization processes in Swedish hospitals—similarities and differences between different modalities

**DOI:** 10.1093/rpd/ncaf115

**Published:** 2026-03-13

**Authors:** Angelica Svalkvist, Maria Hultenmo, Pernilla Jonasson, Patrik Sund, Maria Larsson

**Affiliations:** Department of Biomedical Engineering and Medical Physics, Sahlgrenska University Hospital, Gula stråket 2B, plan 0, SE-413 45 Gothenburg, Sweden; Department of Medical Radiation Sciences, Institute of Clinical Sciences, Sahlgrenska Academy, University of Gothenburg, Gula stråket 2B, plan 3, SE-413 45 Gothenburg, Sweden; Department of Biomedical Engineering and Medical Physics, Sahlgrenska University Hospital, Gula stråket 2B, plan 0, SE-413 45 Gothenburg, Sweden; Department of Biomedical Engineering and Medical Physics, Sahlgrenska University Hospital, Gula stråket 2B, plan 0, SE-413 45 Gothenburg, Sweden; Department of Biomedical Engineering and Medical Physics, Sahlgrenska University Hospital, Gula stråket 2B, plan 0, SE-413 45 Gothenburg, Sweden; Department of Medical Radiation Sciences, Institute of Clinical Sciences, Sahlgrenska Academy, University of Gothenburg, Gula stråket 2B, plan 3, SE-413 45 Gothenburg, Sweden; Department of Biomedical Engineering and Medical Physics, Sahlgrenska University Hospital, Gula stråket 2B, plan 0, SE-413 45 Gothenburg, Sweden; Department of Medical Radiation Sciences, Institute of Clinical Sciences, Sahlgrenska Academy, University of Gothenburg, Gula stråket 2B, plan 3, SE-413 45 Gothenburg, Sweden

## Abstract

The optimization process is an important part of image-based diagnostics and treatments, requiring collaboration among medical physicists, radiologists, physicians, radiographers, nurses, engineers, and vendors. However, such teamwork can be challenging in some departments. In spring 2024, questionnaires were sent to Swedish medical physicists working with radiology, interventional radiology, or surgery facilities using radiological equipment. The surveys included modality-specific questions about optimization processes. The aim was to explore differences in optimization processes between modalities and departments, identify common challenges, and understand factors facilitating effective optimization. Results showed variations in optimization processes across different modalities, where successful optimization processes were harder to achieve for stationary fluoroscopy systems and mobile fluoroscopy systems than for the other modalities. Common challenges included limited time and lack of knowledge about image quality issues, while close collaboration, continuous meetings with focus on optimization, and good communication were mentioned as important factors for obtaining successful optimization processes.

## Introduction

According to International Commission on Radiation Protection (ICRP) Publication 154 [1], there are three different aspects that all contribute to effective optimization processes. The first aspect is related to collaboration within optimization teams, which ideally consist of people representing different professions, in which each included profession needs training and experience regarding how different factors in the imaging chain affect the dose levels and impact the resulting image quality. The second aspect concerns the methodologies used for optimization, which ideally should incorporate patient-specific parameters linked to clinical outcomes, using metrics that reflect image quality and clinical usefulness. The third aspect includes having structured processes with proper documentation established, which helps to ensure that findings from performance evaluations, clinical studies, and patient dose assessments are incorporated in the review of examination protocols.

In the directive from the European Union Council (directive 2013/59/EURATOM [2]), it is stated which radiation protection regulations the member states must employ in their national regulations. According to the directive, the radiation protection shall be based on the principles of justification, optimization, and dose limitation. The 2013/59/EURATOM directive was implemented in Swedish law in 2018 by the Radiation Protection Act [3], which was followed by an ordinance from the Swedish Government [4]. The Swedish Radiation Safety Authority (Strålsäkerhetsmyndigheten, SSM) is assigned the task of compiling regulations based on the Radiation Protection Act and the ordinance of the Swedish Government, which must be followed by all institutions that use ionizing radiation. There are mainly two regulations that concern the use of radiation within a medical context. The first, SSMFS 2018: 1 [5], includes basic regulations that must be followed by all institutions (not only medical institutions) that conduct permit-required activities including ionizing radiation (conduct activities including ionizing radiation that are subject to government authorization). The second one, SSMFS 2018:5 [6], includes advice and regulations specifically for medical exposures.

According to regulation SSMFS 2018:5, the magnitude and radiation dose from a medical diagnostic exposure should be adapted to the individual so that the diagnostic information is obtained using as low a radiation dose as is reasonably possible. The optimization process shall include selection of appropriate modality, verification of diagnostic accuracy, the practical implementation of an examination, quality assurance, and evaluation of clinical practices, and associated patient radiation doses. Additionally, it is stated that all departments conducting activities including medical exposures should have access to two mandated roles: a physician in the relevant field with expert competence [in Swedish: Radiologisk ledningsfunktion (RaLF)] and a medical physicist in the relevant field with expert competence [in Swedish: strålningsfysikalisk ledningsfunktion (SLF)]. Both these roles shall have the necessary competence needed to ensure that medical exposures are conducted in a way that is satisfactory from a radiation protection point of view. Hence, these roles should be adapted to the nature and scope of the activities conducted at the department, have the competence needed to ensure that the medical exposures can be performed in a radiation-safe manner, and provide advice regarding radiation protection of the patients. It is also stated that the RaLF and SLF, together with other personnel involved in medical exposures, should participate in the optimization process to the extent needed from a radiation protection point of view.

The regulation SSMFS 2018:5 also includes information regarding diagnostic reference levels for the most common examinations within each modality. All institutions performing these examinations are obligated to determine and report diagnostic standard levels for the specified examinations. In case the diagnostic standard level exceeds the diagnostic reference level, the cause must be investigated and motivated. In addition, all institutions that perform medical exposures are obligated to report statistics on the annual number of examinations performed and their corresponding average radiation dose for each examination. The reporting of both diagnostic standard levels and annual statistics to the radiation safety authority is made digitally using Dosreg, a website provided by the authority (www.dosreg.ssm.se [7]). In the reporting of diagnostic standard levels, examination protocol settings should also be provided. The information reported is publicly available on the website, with the purpose of facilitating optimization.

In general discussions with medical physicist colleagues at other institutions, the authors of this article have noticed that many experience problems with their optimization processes. Also, the organization of optimization varies between institutions, with some lacking an optimization process altogether. The purposes of the present study were therefore to investigate if there are any differences in optimization processes between different modalities and different departments/institutions in Sweden, identify common challenges, and obtain knowledge regarding important factors that facilitate successful optimization processes.

## Materials and methods

In May 2024, digital surveys, created using Microsoft Forms, were sent to 136 medical physicists in Sweden, working in the field of radiology or interventional radiology. Suitable receivers of the surveys were identified using a public list of medical physicists found on the website for the Swedish Medical Physicist Association, which is the Swedish union for medical physicists. To the authors’ knowledge, the list includes all hospitals in Sweden where medical physicists are employed: 7 university hospitals, 22 county hospitals, and 6 private hospitals. It is, however, known that this is not a complete list of all medical physicists in Sweden due to several different reasons. For example, both membership in the union and having your name on the public list are voluntary. Additionally, the list might not contain correct contact information as it is the individual members’ own responsibility to notify the union of changes so that the list is updated. Therefore, the receivers of the surveys were asked to forward the surveys to other suitable medical physicists in their organization who might not be on the union list. In the information that was sent together with links to the surveys, it was mentioned that different medical physicists in the organization can answer different parts of the surveys, if the institution finds this appropriate. The deadline for responses was set to ~5 weeks from the date the surveys were sent.

The surveys were targeting different modalities: computed tomography (CT), conventional X-ray (including mobile systems), stationary fluoroscopy (not including fluoroscopy using conventional X-ray systems), mobile fluoroscopy, mammography, and dental radiology. The questions in the surveys were chosen to include the three different aspects of developing and improving optimization in an organization that are mentioned in ICRP Publication 154: professionalism, methodology, and processes. Professionalism includes aspects regarding, e.g. professional skills and collaboration, methodology includes aspects regarding, e.g. methods used to monitor and perform optimization, and processes includes aspects concerning e.g. optimization processes and documentation. Hence, the survey included both general questions regarding the optimization process and specific questions regarding, e.g. the use of dose management systems (DMSs) as a tool for optimization, optimization of paediatric protocols, and difficulties/challenges regarding optimization work. In the final question of each survey, the responder was given the opportunity to highlight specific details regarding the optimization process at the institution. The survey is presented in Table 1 (originally, the questions were in Swedish but have been translated to English for publication purposes).

**Table 1:** The surveys as a whole. Note: The questions were originally in Swedish but have been translated into English for publication purposes. SLF = medical physicist in the relevant field with the expert competence [in Swedish: Strålningsfysikalisk ledningsfunktion], RaLF = physician in the relevant field with the expert competence [in Swedish: Radiologisk ledningsfunktion].

**Question**	**Answer alternatives**
1. What type of institution do you represent?	University hospital County hospital Private hospital Other: Free-text
2. How many medical physicists are working with ^*^insert modality^*^ in your institution?	Free-text
3. How many systems of ^*^insert modality^*^ do you have in your institution?	Free-text
4. Which vendors of ^*^insert modality^*^ do you have in your institution?	Free-text
5. Do you have a structured optimization process for ^*^insert modality^*^ examinations? (*A structured optimization process includes, e.g. that there are established routines for how the optimization should be carried out and that there are established groups consisting of people from different professional categories who are involved in the optimization process)*	Yes No Partly
6. If you answered partly, what do you believe is missing?	Free-text
7. Which professions are represented in the optimization of ^*^insert modality^*^ examinations? (multiple answer options possible)	Medical physicist Radiologist Radiology nurse or operating room nurse Licensed practical nurse Medical engineer Vendor Other: Free-text
8. Does the optimization of ^*^insert modality^*^ examinations work equally well in all departments using the modality?	Yes No
9. If you answered ‘No’, describe the differences between the different departments.	Free-text
10. Does the collaboration between RaLF and SLF work well regarding the optimization of ^*^insert modality^*^ examinations?	Yes No
11. If you answered ‘Yes’, describe the collaboration. What is the key to success?	Free-text
12. If you answered ‘No’, describe what is missing/not working	Free-text
13. Which profession initiates optimization efforts of ^*^insert modality^*^ at your institution? (multiple answer options possible)	The department (e.g. due to poor image quality) The medical physicist (e.g. due to high radiation doses) The management The vendor A designated group working on optimization Other: (free-text)
14. Which profession(s) makes decisions about possible adjustments of examination parameters resulting from optimization efforts, and which profession(s) implement the decided adjustments?	Free-text
15. Do you use information from DosReg as a tool in the optimization of ^*^insert modality^*^ examinations?	Yes No
16. If you answered ‘Yes’, which information do you use?	Free-text
17. Do you use a radiation dose management system as a tool in the optimization of ^*^insert modality^*^ examinations?	Yes No
18. If you answered ‘Yes’, which radiation dose management system do you use?	Free-text
19. For how long have your ^*^insert modality^*^ systems been connected to your radiation dose management system?	Less than 2 years 2–5 years More than 5 years
20. Do you obtain sufficient/relevant information for optimization tasks from your radiation dose management system?	Yes No
21. If you answered ‘No’, what information do you believe is missing?	Free-text
22. Do you have optimized paediatric protocols on the systems where paediatric ^*^insert modality^*^ examinations are performed? (Note: question not included for mammography)	Yes, the vendor has installed paediatric protocols Yes, the department has done optimization efforts on paediatric protocols No Partly Not applicable
23. If you answered ‘No’, why not?	Free-text
24. If you answered ‘Partly’, what is missing or what is not optimized?	Free-text
25. Which are currently the most prominent difficulties/challenges concerning the optimization of ^*^insert modality^*^ examinations? (multiple answer options are possible)	Collaboration with the vendor is lacking or insufficient Collaboration/communication between different professions is lacking or insufficient Time for optimization efforts is limited Insufficient level of knowledge among the relevant professions Access to the relevant equipment is limited No access to information regarding radiation doses No access to information regarding image quality issues Lack of access to suitable phantoms Other: Free-text
26. Anything else you would like to highlight regarding the optimization of ^*^insert modality^*^ examinations? *(e.g. examples of successful optimization efforts, description of how you would like the optimization process to work, description of which issues are discussed at optimization meetings, description of patient benefits that you identify as a result of optimization efforts)*	Free-text

^*^Insert modality^*^: computed tomography, conventional X-ray, stationary fluoroscopy, mobile fluoroscopy, mammography, or dental X-ray.

## Results

Please note that, for the types of institutions with less than three respondents, no detailed information is presented as this information might lead to easy identification of the institution. Additionally, the results regarding the number of medical physicists working with each modality (Question 2) are not shown due to inconclusive answers. Some respondents stated the number of people working with the modality, while other respondents stated the corresponding number of full-time positions. With only a few exceptions, all respondents answered all questions in the surveys. Hence, all answers are presented if not stated otherwise.

### Questions 1–4—Responder statistics

In Table 2, the number of responses and detailed information regarding the type of institution, the number of systems, and the number of different vendors (i.e. Questions 1, 3, and 4) are shown for each modality.

**Table 2:** Detailed information regarding the number of responses, the number of systems, and the number of different vendors for the different modalities and the different types of institutions.

**Modality**	**Number of responses**	**Number of systems Mean (range)**	**Number of different vendors** **Mean (range)**
**Computed tomography**	**26**	**8.7 (1–25)**	**1.9 (1–4)**
University hospital	8	17.0 (5–25)	3.0 (1–4)
County hospital	16	6.3 (1–10)	1.4 (1–3)
Private institution	2	^*^	^*^
**Conventional X-ray**	**26**	**15.3 (3–50)**	**3.0 (1–7)**
University hospital	8	22.9 (8–50)	4.1 (2–7)
County hospital	17	5.5 (3–25)	2.7 (1–5)
Private institution	1	^*^	^*^
**Stationary fluoroscopy**	**22**	**8.2 (2–30)**	**2.1 (1–5)**
University hospital	8	15.4 (3–30)	2.8 (1–5)
County hospital	13	5.0 (2–7)	2.0 (1–3)
Private institution	1	^*^	^*^
**Mobile fluoroscopy**	**21**	**22.0 (1–63)**	**4.2 (1–9)**
University hospital	6	28.6 (8–63)	5.3 (2–9)
County hospital	14	17.0 (1–30)	3.6 (1–6)
Private institution	1	^*^	^*^
**Mammography**	**17**	**8.9 (4–40)**	**1.4 (1–3)**
University hospital	6	7.0 (4–10)	1.2 (1–2)
County hospital	10	9.8 (4–40)	1.5 (1–3)
Private institution	1	^*^	^*^
**Dental X-ray**	**20**	**135.6 (2–301)**	**3.3 (1–7)**
University hospital	7	127.5 (2–296)	2.8 (1–5)
County hospital	12	133.9 (2–301)	3.7 (1–7)
Public dental care	1	^*^	^*^

^*^Less than three respondents, why detailed data are not shown. Bold text: Results summarized for all institutions. Plain text: Results for each type of institution.

For all modalities, except mammography and dental X-ray, the university hospitals have both a larger mean number of systems and a larger mean number of different vendors than the county hospitals. The variations in the number of systems and the number of vendors are, however, substantial both for university hospitals and county hospitals. Data from private institutions and the institution for public dental care are not shown due to less than three respondents.

### Questions 5–7—The optimization process

The results for the question regarding the structured optimization process (Question 5) is shown in Table 3. For CT and conventional X-ray, the majority answered that they have structured optimization processes, while the most common answer for both stationary and mobile fluoroscopy was ‘partly’. For mammography and dental X-ray, the answers were more equally distributed between the answering alternatives.

**Table 3:** The distribution of answers to question number 5 for different modalities and the type of institution.

**Modality**	**Do you have a structured optimization process?**		
	**Yes**	**Partly**	**No**
**Computed tomography**	**18**	**5**	**1**
University hospital	7	1	0
County hospital	11	4	1
**Conventional X-ray**	**16**	**7**	**2**
University hospital	7	1	0
County hospital	9	6	2
**Stationary fluoroscopy**	**4**	**11**	**6**
University hospital	2	5	1
County hospital	2	6	5
**Mobile fluoroscopy**	**2**	**11**	**7**
University hospital	1	3	2
County hospital	1	8	5
**Mammography**	**6**	**5**	**5**
University hospital	2	2	2
County hospital	4	3	3
**Dental X-ray**	**6**	**6**	**7**
University hospital	3	1	3
County hospital	3	5	4

Bold text: Results summarized for all institutions. Plain text: Results for each type of institution.

Those who answered ‘partly’ described various deviations from a structured optimization process (Question 6), i.e. lack of routines, routines exist but are not followed, no regular meetings, not structured meetings/methods, no defined working groups, and only structured optimization processes for some of the departments using the X-ray modality.

The results for the question regarding which professions are represented in the optimization process (Question 7) are shown in Table 4. For all modalities, a medical physicist is involved in the optimization process. For CT, conventional X-ray, stationary fluoroscopy, and mammography, radiologists and radiology nurses/operating room nurses are almost always included in the process. For mobile fluoroscopy, a licensed practical nurse is more commonly involved in the optimization process than a radiology nurse or an operating room nurse. Quite a few respondents have answered that the vendor is included in the optimization process. This seems to be most common within stationary fluoroscopy. Please note that for dental X-ray, only 17 of the 19 respondents from university hospitals and county hospitals answered the question.

**Table 4:** The distribution of answers regarding question number 7 for different modalities and the type of institution. For dental X-rays, only 17 of the 19 respondents answered the question.

**Modality**	**Which professions are represented in the optimization process?**					
	**Medical physicist**	**Radiologist**	**Radiology nurse or operating room nurse**	**Licensed practical nurse**	**Medical engineer**	**Vendor**
**Computed tomography**	**24**	**24**	**24**	**2**	**3**	**11**
University hospital	8	8	8	0	1	2
County hospital	16	16	16	2	2	9
**Conventional X-ray**	**24**	**24**	**24**	**2**	**1**	**7**
University hospital	8	8	8	1	0	1
County hospital	18	18	18	1	1	6
**Stationary fluoroscopy**	**21**	**17**	**18**	**3**	**3**	**13**
University hospital	8	7	8	1	1	5
County hospital	13	10	10	2	2	8
**Mobile fluoroscopy**	**20**	**4**	**8**	**11**	**6**	**9**
University hospital	6	1	2	4	2	4
County hospital	14	3	6	7	4	5
**Mammography**	**16**	**16**	**13**	**0**	**3**	**7**
University hospital	6	6	5	0	2	2
County hospital	10	10	8	0	1	5
**Dental X-ray**	**17**	**12**	**6**	**0**	**3**	**4**
University hospital	7	6	3	0	2	2
County hospital	10	6	3	0	1	2

Bold text: Results summarized for all institutions. Plain text: Results for each type of institution.

In free text, other professions mentioned were cardiologists, orthopaedists, and other surgical sub-professions (for stationary fluoroscopy) and dentists and dentist nurses (for dental X-ray).

### Questions 8–9—Optimization processes in different departments

For most modalities, a majority of respondents answered that the optimization process is working equally well in all departments that use the modality (Question 8). The only exception is found for stationary fluoroscopy where the majority of the respondents have answered that there are differences between the departments (see Table 5).

**Table 5:** The distribution of answers to question number 8 for different modalities and the type of institution.

**Modality**	**Does the optimization process work equally well in all departments that use the modality?**	
	**Yes**	**No**
**Computed tomography**	**19**	**5**
University hospital	6	2
County hospital	13	3
**Conventional X-ray**	**17**	**8**
University hospital	6	2
County hospital	11	6
**Stationary fluoroscopy**	**8**	**13**
University hospital	1	7
County hospital	7	6
**Mobile fluoroscopy**	**11**	**9**
University hospital	1	5
County hospital	10	4
**Mammography**	**15**	**1**
University hospital	6	0
County hospital	9	1
**Dental X-ray**	**13**	**6**
University hospital	5	2
County hospital	8	4

Bold text: Results summarized for all institutions. Plain text: Results for each type of institution.

The most commonly mentioned causes for differences (Question 9) are various levels of interest and commitment, different levels of personnel resources, and time dedicated to tasks related to the optimization process. Other reasons mentioned are various geographical distances to the different departments and organisational differences between the departments.

### Questions 10–12—The collaboration between RaLF and SLF

The collaboration between the RaLF and SLF regarding optimization processes (Question 10) is, in general, well functioning for most modalities. The exception is found for mobile fluoroscopy where the majority have answered that the collaboration is not well functioning, see Table 6. According to the respondents who answered that the collaboration is well-functioning, the mentioned reasons for the successful collaboration (Question 11) were good communication between the professions, short geographical distances, regular meetings, clear directives from the management, and continuous communication regarding the benefits of well-functioning optimization processes for both patients and the working environment. Those respondents who answered that the collaboration is of inferior standard (Question 12) expressed different reasons, among them, poor communication, lack of time, and personal resources, lack of established working routines, lack of interest from the management, and lack of interest from RaLF (in some cases, RaLF is not primarily working with the modality). However, many of the respondents mention that RaLF and SLF are not involved in practical optimization but instead work on a more organisational level by, e.g. formulating routines included in the optimization process.

**Table 6:** The distribution of answers to question number 10 for different modalities and the type of institution. RaLF = physician in the relevant field with the expert competence [in Swedish: Radiologisk ledningsfunktion], SLF = medical physicist in the relevant field with the expert competence [in Swedish: Strålningsfysikalisk ledningsfunktion].

**Modality**	**Does the collaboration between the RaLF and the SLF work well regarding the optimization process?**	
	**Yes**	**No**
**Computed tomography**	**20**	**4**
University hospital	6	2
County hospital	14	2
**Conventional X-ray**	**19**	**6**
University hospital	6	2
County hospital	13	4
**Stationary fluoroscopy**	**15**	**6**
University hospital	6	2
County hospital	9	4
**Mobile fluoroscopy**	**8**	**12**
University hospital	2	4
County hospital	6	8
**Mammography**	**10**	**6**
University hospital	3	3
County hospital	7	3
**Dental X-ray**	**16**	**3**
University hospital	7	0
County hospital	9	3

Bold text: Results summarized for all institutions. Plain text: Results for each type of institution.

### Questions 13–14—Initiation of optimization efforts and implementation of protocol changes

According to the respondents, optimization efforts are commonly initiated by either the department or the medical physicist (Question 13), see Table 7. For CT and conventional X-ray, it is also common that the optimization effort is initiated by a designated group. However, it is more common that the vendor initiates the optimization efforts for stationary fluoroscopy than for the other modalities.

**Table 7:** The distribution of answers to question number 13 for different modalities and the type of institution.

**Modality**	Which profession initiates optimization efforts of ^*^insert modality^*^ at your institution?				
	**The department (e.g. due to poor image quality)**	**The medical physicist (e.g. due to high radiation doses)**	**The management**	**The vendor**	**A designated group working on optimization**
**Computed tomography**	**23**	**23**	**0**	**2**	**16**
University hospital	8	8	0	0	6
County hospital	15	15	2	0	10
**Conventional X-ray**	**24**	**25**	**1**	**4**	**14**
University hospital	8	8	0	2	4
County hospital	16	17	1	2	10
**Stationary fluoroscopy**	**20**	**10**	**0**	**7**	**7**
University hospital	8	8	0	3	5
County hospital	12	12	0	4	2
**Mobile fluoroscopy**	**19**	**16**	**0**	**0**	**3**
University hospital	6	5	0	0	1
County hospital	13	11	0	0	2
**Mammography**	**15**	**14**	**2**	**1**	**4**
University hospital	6	6	2	0	1
County hospital	9	8	0	1	3
**Dental X-ray**	**16**	**16**	**0**	**1**	**2**
University hospital	7	6	0	0	1
County hospital	9	10	0	1	1

Bold text: Results summarized for all institutions. Plain text: Results for each type of institution. *insert modality* : computed tomography, conventional X-ray, stationary fluoroscopy, mobile fluoroscopy, mammography, or dental X-ray.

Regarding Question 14, most respondents, regardless of modality, answered that the decisions about adjustments of examination parameters are made in collaboration between radiologists, medical physicists, and radiographers. For stationary fluoroscopy and mobile fluoroscopy, however, it is more common that a representative from the vendor implements changes in examination protocols.

### Questions 15–16—Information from the Dosreg website

For CT and conventional X-ray, a majority of respondents answered that they use information from the Dosreg website as a tool in the optimization process (Question 15). For stationary fluoroscopy, mammography, and dental X-ray, about half of the respondents gave a positive answer, whereas only a few positive answers were given for mobile fluoroscopy (see Table 8).

**Table 8:** The distribution of answers to question number 15 for different modalities and the type of institution.

**Modality**	**Do you use information from Dosreg as a tool in your optimization process?**	
	**Yes**	**No**
**Computed tomography**	**18**	**6**
University hospital	4	4
County hospital	14	2
**Conventional X-ray**	**17**	**8**
University hospital	4	4
County hospital	13	4
**Stationary fluoroscopy**	**10**	**11**
University hospital	3	5
County hospital	7	6
**Mobile fluoroscopy**	**2**	**18**
University hospital	0	6
County hospital	2	12
**Mammography**	**9**	**7**
University hospital	2	4
County hospital	7	3
**Dental X-ray**	**10**	**9**
University hospital	4	3
County hospital	6	6

Bold text: Results summarized for all institutions. Plain text: Results for each type of institution.

Most of the respondents answered that they use the information in Dosreg mainly to compare the examination parameters and resulting diagnostic standard levels with other institutions that have systems of the same model (Question 16).

### Questions 17–21—Dose management systems

The results regarding Questions 17 and 19 are shown in Table 9. For all modalities, except dental X-ray, DMSs are commonly used as a tool in the optimization process (Question 17). For CT, conventional X-ray, and stationary fluoroscopy, most respondents answered that their systems have been connected to the DMS for >5 years (Question 19). The respondents who answered that they use a DMS as a tool in the optimization process also answered a question regarding the information obtained from the DMS (Question 20). A relatively large number of respondents answered that the information obtained from the DMS is not always sufficient for optimization tasks (Table 10). Lacking information (Question 21) includes, e.g. dose modulation settings, reconstruction parameters, image postprocessing settings, patient height and weight, and information that can help to distinguish multi-organ examinations from single-organ examinations. Please note that detailed responses regarding Question 18 (Which dose management system do you use?) are not shown. However, for all modalities, the Sectra DoseTrack™ is the most commonly used DMS.

**Table 9:** The distribution of answers to question numbers 17 and 19 for different modalities and the type of institution.

**Modality**	**Do you use a dose management system as a tool in your optimization process?**		**For how long have your systems been connected to your dose management system? (years)**		
	**Yes**	**No**	**< 2**	**2–5**	**> 5**
**Computed tomography**	**21**	**3**	**2**	**5**	**14**
University hospital	8	0	1	1	6
County hospital	13	3	1	4	8
**Conventional X-ray**	**20**	**5**	**1**	**5**	**14**
University hospital	8	0	1	1	6
County hospital	12	5	0	4	8
**Stationary fluoroscopy**	**19**	**2**	**2**	**3**	**14**
University hospital	8	0	1	1	6
County hospital	11	2	1	2	8
**Mobile fluoroscopy**	**13**	**7**	**4**	**5**	**4**
University hospital	4	2	2	1	1
County hospital	9	5	2	4	3
**Mammography**	**14**	**2**	**2**	**5**	**7**
University hospital	6	0	1	2	3
County hospital	8	2	1	3	4
**Dental X-ray**	**5**	**14**	**1**	**2**	**2**
University hospital	3	4	0	1	2
County hospital	2	10	1	1	0

Bold text: Results summarized for all institutions. Plain text: Results for each type of institution.

**Table 10:** The distribution of answers to question number 20 for different modalities and type of institution.

**Modality**	**Do you obtain sufficient/relevant information for optimization tasks from your dose management system?**	
	**Yes**	**No**
**Computed tomography**	**13**	**8**
University hospital	7	1
County hospital	6	7
**Conventional X-ray**	**16**	**4**
University hospital	8	0
County hospital	6	4
**Intervention**	**9**	**10**
University hospital	3	5
County hospital	6	5
**Mobile X-ray**	**9**	**4**
University hospital	4	0
County hospital	5	4
**Mammography**	**8**	**6**
University hospital	4	2
County hospital	4	4
**Dental X-ray**	**3**	**2**
University hospital	1	2
County hospital	2	0

Bold text: Results summarized for all institutions. Plain text: Results for each type of institution.

### Questions 22–24—Paediatric protocols

The results from the question regarding examination protocols for paediatric examinations (Question 22) are shown in Table 11. For CT only, 11 of 26 respondents answered this question and only 1 from a university hospital. For the other modalities, the majority have optimized paediatric protocols, either protocols implemented by the vendor or protocols optimized by the departments themselves. Those who answered ‘no’ (Question 23) described the reason as being that the paediatric examinations are very few. Those who answered ‘partly’ described various reasons (Question 24), e.g. protocols are copied from other institutions, all protocols at the paediatric radiology department are optimized but not all at other radiological departments, and protocols for adults are used together with automatic dose modulation.

**Table 11:** The distribution of answers to question number 22 for different modalities and the type of institution.

**Modality**	**Do you have optimized protocols for paediatric examinations?**				
	**Yes, the vendor has implemented paediatric protocols**	**Yes, the department has done optimization efforts on paediatric protocols**	**No**	**Partly**	**Not applicable**
**Computed tomography**	**4**	**5**	**0**	**2**	**0**
University hospital	0	0	0	1	0
County hospital	4	5	0	1	0
**Conventional X-ray**	**4**	**16**	**0**	**5**	**0**
University hospital	0	7	0	1	0
County hospital	4	9	0	4	0
**Stationary fluoroscopy**	**10**	**2**	**3**	**3**	**3**
University hospital	5	1	0	1	1
County hospital	5	1	3	2	2
**Mobile fluoroscopy**	**7**	**1**	**5**	**5**	**2**
University hospital	2	1	1	1	1
County hospital	5	0	4	4	1
**Mammography**	Not applicable				
University hospital					
County hospital					
**Dental X-ray**	**7**	**8**	**2**	**2**	**0**
University hospital	3	4	0	0	0
County hospital	4	4	2	2	0

Bold text: Results summarized for all institutions. Plain text: Results for each type of institution. Note: For CT, only 11 of 26 respondents answered this question.

### Question 25—Difficulties and challenges concerning the optimization process

The results from question 25 are shown in Table 12. By far, the most frequently mentioned challenge overall was ‘Not enough time for optimization’, followed by ‘Access to the relevant equipment is limited’ and ‘no access to information regarding image quality issues’. The former was more common for CT, conventional X-ray, and stationary fluoroscopy than for the other modalities. For stationary fluoroscopy and mobile fluoroscopy, the second most common challenge was ‘Collaboration/communication between different professions is lacking or insufficient’, a challenge that was less frequently mentioned for the other modalities. The challenge ‘Insufficient level of knowledge among the relevant professions’ was more common for mobile fluoroscopy than for the other modalities. Other challenges mentioned were, e.g. inadequate or lacking external protocol management software (CT and conventional X-ray), missing information regarding patient height and weight (conventional X-ray), no common understanding of the term ‘optimization’ (conventional X-ray), systems with increased complexity and limited access to protocol adjustments without vendor assistance (stationary fluoroscopy), the geographical location and insufficient optimization processes (mobile fluoroscopy), limited access to radiologists (mammography), and low priority for optimization (dental X-ray).

**Table 12:** The distribution of answers to question number 25 (the most prominent difficulties/challenges concerning the optimization) for different modalities and the type of institution.

**Modality**	**Time for optimization efforts is limited**	**Access to the relevant equipment is limited**	**No access to information regarding image quality issues**	**Collaboration/communication between different professions is lacking or insufficient**	**Lack of access to suitable phantoms**	**Collaboration with vendor is lacking or insufficient**	**Insufficient level of knowledge among the relevant professions**	**No access to information regarding radiation doses**
**Computed tomography**	**19**	**15**	**7**	**5**	**5**	**2**	**1**	**1**
University hospital	6	6	3	2	1	1	0	0
County hospital	13	9	4	3	4	1	1	1
**Conventional X-ray**	**22**	**13**	**10**	**9**	**5**	**2**	**2**	**1**
University hospital	7	4	3	4	3	1	0	0
County hospital	15	9	7	5	2	1	2	1
**Stationary fluoroscopy**	**19**	**10**	**10**	**12**	**8**	**5**	**4**	**2**
University hospital	6	5	4	4	6	2	0	1
County hospital	13	5	6	8	2	3	4	1
**Mobile fluoroscopy**	**17**	**5**	**10**	**14**	**5**	**4**	**9**	**4**
University hospital	3	1	2	3	3	1	2	1
County hospital	14	4	8	11	2	3	7	3
**Mammography**	**11**	**3**	**4**	**2**	**5**	**1**	**1**	**0**
University hospital	5	1	4	0	3	0	0	0
County hospital	7	2	1	2	2	1	1	0
**Dental X-ray**	**11**	**1**	**4**	**2**	**8**	**5**	**2**	**3**
University hospital	4	1	2	1	4	2	0	1
County hospital	7	0	2	1	4	3	2	2
Total	99	47	45	44	36	19	19	11

Bold text: Results summarized for all institutions. Plain text: Results for each type of institution.

### Question 26—General comments/reflections

#### Computed tomography

Many respondents mentioned the fact that it is beneficial to have many systems of the same model as this enables the possibility to optimize the protocols on one reference system and then implement the results on the other systems. The use of software that enables adjustments of examination protocols without direct access to the CT system was mentioned as a prerequisite for being able to make changes to the protocols without interfering with the ongoing patient examinations. One facility mentioned that they have developed a standard for indexing the different CT examinations, which has enabled coherent indexing of each specific type of examination and examination protocol.

One respondent mentioned that the regular meetings that previously were dedicated towards optimization have now been converted into modality meetings in which all aspects concerning the modality are discussed. Consequently, less time is now spent on optimization-specific questions. In general, many respondents mention shortness of staff as a problem for a successful optimization process. One respondent stated that great things can be accomplished if there is access to a radiologist who is also a PhD student and passionate about optimizing protocols. However, as concluded by the respondent; ‘…that rarely happens’ [‘…det är sällan det händer.’].

#### Conventional X-ray

Also, for conventional X-ray, the benefits of having many systems of the same model were mentioned by several respondents. However, for this modality, many respondents also mentioned that there is a lack of structured and proactive optimization processes; instead, optimization efforts occur only when problems with the examinations become urgent. One of the respondents has written ‘On the few occasions we have been given the permission to spend time on optimisation, we manage to work efficiently and make important changes’. [‘Vid de enstaka tillfällen vi fått tillåtelse att lägga tid på optimering lyckas vi arbeta effektivt och utföra viktiga förändringar’.]

One of the respondents writes that any technology that makes things simpler also tends to make people ‘more stupid’ in the long run. The respondent elaborates that, due to all the automatic processes included in modern X-ray systems, the X-ray personnel might manage to obtain images with decent quality without considering the exposure parameters. However, the understanding of all the background processes affecting the outcome of a radiological examination is lost along the way, meaning that the personnel eventually become unable to reflect upon the possible reasons for suboptimal image quality or high patient doses. The respondent believes that structured optimization processes could be one way to counteract this phenomenon.

According to one responder, they would benefit from access to additional phantoms, especially anthropomorphic phantoms of children. Unfortunately, such phantoms are expensive, and many institutions are unable to finance the purchase themselves. The responder suggests that one solution could be to establish a national phantom bank from which suitable phantoms could be borrowed or rented when needed.

#### Stationary fluoroscopy

For stationary fluoroscopy, a couple of respondents mentioned that optimization efforts in their institutions rarely include adjustments of examination parameters. Instead, the optimization efforts are more focused on ensuring that the operators work in the most radiation-safe manner possible. According to these respondents, this approach often ‘saves’ more radiation dose than making minor adjustments to the examination protocols.

Many of the respondents also agree upon the fact that the institutions are dependent on the vendors for optimization efforts, due to restrictions in the system software. The experience is that optimization efforts are mainly done upon delivery of new systems, but problems with image quality are usually discovered after the system has been in clinical use for some time. One of the respondents suggests that it might be beneficial to, in the future, stipulate requirements regarding vendor-supplied resources for optimization efforts already during the procurement of systems.

#### Mobile fluoroscopy

Similar to stationary fluoroscopy, the optimization efforts for mobile fluoroscopy mainly include education of staff so that they operate the systems in a radiation-safe manner. Education, training, and practical radiation protection exercises are mentioned as important and of great value to ensure that the equipment is correctly used and results in reductions of fluoroscopy times and radiation doses.

One of the respondents answered that the medical physicists generally have less insight into how the systems are used in, e.g. surgery departments, and that the personnel handling the systems have a low interest in working with optimization efforts. One opinion mentioned in the responses is that it would be beneficial if the vendors could take a greater responsibility to unify settings over different sites. The experience is that there are unnecessarily large differences in exposure settings between different sites.

#### Mammography

In mammography, one of the respondents answered that they try to minimize adjustments of examination parameters during the lifetime of the system as the purpose of mammography screening is facilitated by similar imaging settings for recurrent examinations. Instead, the respondent tries to actively participate during installation of new systems in order to make sure that the protocol settings ensure similar image quality as the older systems. It is, however, also mentioned that some of the older systems have limitations that prohibit the ability to adjust and optimize the examination protocols.

#### Dental X-ray

In dental X-ray, the respondents express that the large amount of different system models and sensors makes it difficult to optimize the examinations performed using intraoral systems. Additionally, it can also be difficult to determine requirements for acceptable image quality for specific clinical referrals. However, a couple of respondents report that they have developed a method for determining exposure tables, which they have found to be a valuable tool for achieving consistent and good image quality for intraoral systems.

Another problem that is mentioned by the respondents regarding dental X-ray is that the Digital Imaging and Communications in Medicine (DICOM) information, in many cases, is of inferior quality, as detailed information regarding, e.g. field sizes might not be reported.

Most respondents agree upon the fact that more optimization efforts are performed on cone-beam CT systems and panoramic systems than on intraoral systems.

## Discussion

According to ICRP Publication 154 [1], the initial stages of the optimization process often consist of discrete activities, such as practical performance testing, protocol development, and patient dose audits. The different professions will have different tasks in the optimization process. In general, all professions could be involved in, e.g. establishing routines and documentation of the optimization process. Regarding specific practical tasks, the different professions might have different assignments. For example, medical physicists could perform measurements to test equipment functionality and provide information of radiation dose levels, radiologists (or other physicians evaluating the images) could provide information regarding the obtained image quality and decide if the image quality is sufficient for the specific clinical task, and the radiology nurse (or other nurses) can provide input on practical details in the examination situation that might affect the radiation doses and image quality, such as, e.g. patient positioning and timing of contrast agent infusion. For some modalities, participation from the medical engineers and specialists from the vendors is also important to solve problems connected to radiation dose and image quality. However, to ensure coherence and sustainability, these activities should be integrated into a structured framework that facilitates a consistent execution by the various professions within the imaging team. To reach successful optimization processes, collaboration between the different professions is required. It is also important that professionals obtain suitable training and experience regarding how different factors in the imaging chain affect the dose levels and impact the image quality. In ICRP Publication 154, the development of optimization processes is accomplished in four different steps (or levels) reaching from D to A, where each layer represents a more evolved process. The first layer, called ‘Level D – Preliminary’, represents the early stages needed to finally reach an optimal optimization process, while the highest level, called ‘Level A – Advanced’, represents the optimal optimization process, which is described as an optimization process that is structured and involves regular collaboration in optimization teams. Additionally, the review of image protocols is based on objective data regarding image quality and dose, and dosimetry is based on patient-specific parameters.

Many of the respondents consider their institutions to have structured or partly structured optimization processes (Table 3). However, there are differences between different modalities, different institutions, and different departments where the modalities are used. For example, for CT and conventional X-ray, a majority of the respondents consider their institutions to have a structured optimization process, while this is less common for the other modalities and especially for stationary fluoroscopy and mobile fluoroscopy (Table 3). Opposite to CT and conventional X-ray, which, to a large extent, is installed mainly in the radiological departments where the examinations are more standardized and performed mainly by radiology nurses, the stationary and mobile fluoroscopy systems are found in departments with different specialties, such as, e.g. neurology, cardiology, orthopaedics, vascular surgery, and general surgery, and different organizations and managements. This might partly explain the obtained results. Additionally, the performed fluoroscopic procedures are complex and are, to a large extent, dependent on patient-specific parameters, which is why optimization of image quality is often performed live during the clinical procedures. For these reasons, it might be more challenging to implement a structured optimization process in these departments.

The diverse nature of the departments that use stationary and mobile fluoroscopy systems might probably also explain the results from question 8, regarding whether the optimization process works equally well in all departments that use the modality (Table 5). The different departments might have different organizations, managements, and resources that could affect their possibilities to establish well-functioning optimization processes.

For mammography and dental X-ray, a relatively large proportion of the respondents answered that they do not have a fully structured optimization process. For these modalities, the reason might be found in the free-text answers to Question 25. For example, for mammography, it is mentioned by some of the respondents that there are limitations in many systems that constrain the possibilities for the users to alter the exposure parameters. Additionally, it is also mentioned that, since the screening situation benefits from similar image quality for subsequent examinations of the same patient, protocol changes are made restrictively during the lifetime of the systems. Instead, more efforts are focused on optimizing the protocols on a system during the installation phase. For dental X-ray, many respondents answered that optimization is not prioritized for this modality, especially not for intraoral systems, which might also limit the motivation to work on obtaining a structured optimization process.

The results from the present study show that medical physicists, radiologists (or other physicians), and radiology nurses or operating room nurses, to a large extent, participate in the optimization process (Table 4). This is especially true for CT and conventional X-ray and can be due to the fact that these modalities are mainly located in radiology departments, where all staff involved in the work belong to the same organization. For stationary fluoroscopy, the answers indicate that it is more common that the vendor is involved in the practical optimization efforts than for the other modalities. This was also reflected by the answers to Questions 14 and 25. Regarding Question 14, many of the respondents answered that representatives from the vendors implement protocol changes for the stationary and mobile fluoroscopy systems. In the free-text answers to Question 25, the respondents elaborated upon the fact that the possibilities to optimize the exposure parameters of the stationary fluoroscopy systems are limited due to restrictions in the systems. Hence, in many cases, it seems like the institutions are dependent on the vendors to make necessary changes in the exposure parameters. Given the fact that these systems are complex, this might be a necessary limitation. However, this also requires that the vendors have enough employees to meet the needs of the hospitals regarding optimization efforts. The suggestion from one of the respondents, that requirements regarding vendor resources for optimization efforts should be included in the procurement, could be beneficial for both the hospitals and the vendors. The hospitals would thereby be assured that they get the assistance they need from the vendors, while the vendors can be prepared for the demands from their customers and make sure that they have enough resources to meet these demands. For mobile fluoroscopy, it was significantly more common that assistant nurses participated in the optimization process compared to the other modalities. This is probably a reflection of the organization in the surgery departments where these systems usually operate.

In the previous regulations from the Swedish Radiation Safety Authority (SSMFS 2008:31 [8]), it was stated that RaLF should be a licensed physician with specialist competence within medical radiology, neuroradiology, or paediatric radiology. Hence, for other departments than the radiology departments, the authors’ experience is that the assigned RaLF sometimes might have been just a name on a paper that had little knowledge about the ongoing activities in the department. However, in the recent regulations (SSMFS 2018:5 [6]), this requirement has been changed. While RaLF for radiology departments still needs to be a physician with specialist competence within radiology, RaLF for other departments using X-ray shall now be a physician with specialist competence in radiology or a specialist competence in another area relevant to the activities of the department. This has created the possibility to assign RaLF to a physician who is involved in the medical exposures in each department. However, when RaLF is a physician with specialist competence in a different area than radiology, additional education regarding justification, optimization, and radiation protection might be needed as these physicians may lack suitable knowledge regarding these aspects. Therefore, there is probably still a large proportion of departments that do not have access to a RaLF that has sufficient knowledge about the medical exposures conducted within the department, which might partly explain the fact that the majority of the respondents answer that the collaboration between the RaLF and the SLF is not well-functioning for mobile fluoroscopy (Table 6).

Collection and evaluation of both exposure and image data are important parts of the optimization process. This task is facilitated by the use of DMS, which today has become an important tool for quality assurance and optimization of patient exposures. Loose *et al*. [9] have, on behalf of the European Society of Radiology, performed a survey regarding the use of DMS in Europe. It was found that the most commonly reported problem with the use of DMS was connected to an inadequate harmonization of protocol names, which prohibits efficient evaluations of exposure data. However, according to the results of the present survey, nonharmonized protocol names do not seem to be the only problem connected to the use of DMS. A relatively large number of respondents answered that the information obtained from the DMS is not fully sufficient/relevant for optimization tasks. According to the publication ‘Safety Reports Series 112: Patient Radiation Exposure Monitoring in Medical Imaging’ by the International Atomic Energy Agency (IAEA) [10], the information available from imaging equipment varies between different systems. According to the report from the IAEA, this is because the DICOM standard is insufficient as it may be open to interpretation. Additionally, some of the information remains optional for the vendors. Hence, when using DMS as a tool in the optimization process, one must be aware of the limitations in the DMS software and the fact that some data might be missing and that some of the obtained data might be erroneous.

Regarding Question 25 (Table 12), the fact that lack of time was the most reported challenge concerning the optimization process may not be surprising. Nevertheless, given the fact that optimization is included in the European Union Council in directive 2013/59/EURATOM [2], and thereby implemented in Swedish law [3], one can argue that optimization should be prioritized in all departments using X-ray equipment. However, in some cases, the management of departments might not be fully aware of the regulations. This could be solved by implementing courses with focus on the regulations concerning radiation protection that are mandatory for personnel managing departments where radiation is used for imaging. The problems connected to access to the relevant equipment could possibly be reduced in the future, when software enabling protocol adjustment without physical access to the X-ray systems becomes more common. Another commonly mentioned challenge in the survey is insufficient or a lack of collaboration/communication between different professions. This is emphasized by the fact that many respondents answered that good communication and regular meetings are key to successful collaboration. Solving the problem with insufficient communication might result in many positive outcomes. For example, with well-functioning communication and regular meetings, the problem associated with limited access to information regarding image quality issues might also be automatically solved.

There are some limitations to the present study. For example, even though the questions in the survey were chosen to include the three different aspects of developing and improving the optimization process in an organization that are mentioned in ICRP Publication 154 [1] (professionalism, methodology, and processes), the questions did not fully cover these different aspects. Several more questions were discussed by the authors, but to obtain a reasonable response time for the respondents to answer the surveys, it was decided that the number of questions needed to be limited. Another limitation connected to the questions is the fact that the discrimination between optimization efforts and optimization process was not clearly defined for all questions in the survey. Instead, most questions only referred to ‘optimization’ instead of, e.g. ‘optimization process’, see Table 1. Hence, the respondents might have answered the questions differently depending on their interpretation of the question, e.g. if they interpreted the questions to regard a specific part of the optimization process (e.g. specific optimization efforts) or the entire optimization process. However, based on the free-response answers given by the respondents, it seems like the majority of the respondents have interpreted the questions to cover the entire optimization process. Perhaps the formulation of Question 5, where the term ‘structured optimization process’ was defined, helped to set the minds of the respondents to consider the entire optimization process also for the remaining questions in the surveys. However, in retrospect, Question 25 should ideally also have included answer alternatives reflecting challenges connected to an insufficient optimization process, e.g. answer alternatives such as ‘absence of working routines’ or ‘absence of documentation’. Some of the respondents have, however, mentioned challenges connected to the optimization process in their free-text responses to Question 25, e.g. no common understanding of the term ‘optimization’ and insufficient optimization processes.

The survey was only sent to Swedish medical physicists. Different answers to the questions might be obtained if the surveys were sent to medical physicists in other countries or if other professions answered the same questions. The surveys were sent to 136 medical physicists, employed by a total of 7 university hospitals, 22 county hospitals, and 6 private hospitals. However, in Sweden, medical physicists employed at a specific hospital are in some cases responsible for the imaging equipment also in other hospitals. As the answers in the surveys were anonymous, the authors have no knowledge about which institutions are represented in the results. It is also not possible to evaluate if some of the respondents for each modality represent the same institution or if the surveys were answered by individuals or in collaboration with all medical physicists working with the modality in each institution. For example, in Sweden, there are currently seven university hospitals. However, responses from eight university hospitals were received for CT, conventional X-ray, and stationary fluoroscopy. This could be due to the fact that Sweden previously had eight university hospitals. In 2010, two of the university hospitals were organizationally merged into one university hospital, while the hospitals geographically remained separated. If medical physicists from these two hospitals answered the surveys separately, this could explain the total of eight respondents from university hospitals. Finally, all hospitals might not have access to all the different modalities, which might explain why fewer responses were received for some of the modalities than for others (Table 2).

According to ICRP publication 154 [1], optimization of medical imaging procedures should not mainly be focused on reducing the radiation doses. Instead, the focus should be on obtaining the required clinical information from the imaging situation while keeping the radiation exposure of the individual patients as low as possible. Hence, optimizing specific imaging protocols with the main goal of reducing the radiation exposure is not always the correct approach to lower the patient exposure. Instead, the focus should be on selecting the optimal type of examination for each individual patient in order to avoid unnecessary or inadequate examinations and obtain image quality that is adequate for the clinical purpose. Hence, future evaluations regarding the aspect of justification of the exposures, e.g. how it is ensured that the correct examination is performed for a specific patient, should be considered.

## Conclusions

According to the answers from the conducted survey, different modalities and departments have different prerequisites and challenges regarding the optimization processes of radiological examinations. The most reported challenges in the survey are lack of time and lack of knowledge about image quality problems. Obtaining well-functioning optimization processes seems to be more challenging for stationary and mobile fluoroscopy systems than for other modalities. Regular meetings focusing on optimization and well-functioning communication within the optimization group are mentioned by the respondents as key factors for successful optimization processes.

## Notes

Contribution for special issue: OXMI 2025
